# Sexual behavior and its association with persistent oral lesions: analysis of the POP-Brazil study

**DOI:** 10.1007/s00784-020-03407-0

**Published:** 2020-06-25

**Authors:** Amanda Ramos da Cunha, Marina Bessel, Fernando Neves Hugo, Flávia Moreno Alves de Souza, Gerson Fernando Mendes Pereira, Eliana Márcia Da Ros Wendland

**Affiliations:** 1 Federal University of Rio Grande do Sul, Faculty of Dentistry, Rua Ramiro Barcelos 2492, Porto Alegre, Rio Grande do Sul 90035-003 Brazil; 2 Hospital Moinhos de Vento. Rua Ramiro Barcelos 910, Porto Alegre, Rio Grande do Sul 90035-001 Brazil; 3 Institute of Health Metrics and Evaluation, University of Washington, 2301 Fifth Avenue, Suite 600, Seattle, WA 98121 USA; 4 Department of STI, AIDS and Viral Hepatitis, Ministry of Health, Esplanada dos Ministérios, Bloco G, Brasília, Distrito Federal 70058-900 Brazil; 5 Federal University of Health Science of Porto Alegre, Rua Sarmento Leite 245, Porto Alegre, Rio Grande do Sul 90050-170 Brazil

**Keywords:** Sexual behavior, Oral health, Oral mucosa, Human papillomavirus, Sexually transmitted diseases

## Abstract

**Objectives:**

To investigate whether the presence of persistent self-reported oral lesions (PSOLs) is associated with sexual behaviors and with the presence of sexually transmitted infections (STIs) in individuals aged 16–25 years in the state capitals of Brazil.

**Materials and Methods:**

Data from the POP-Brazil study were analyzed. An association analysis was performed by Poisson regression with the presence of PSOLs as the outcome. The exposure variables were the age at first sexual intercourse, the number of partners, oral sex practice, and aspects of condom use for model 1, and the presence of a self-reported STI or a positive rapid test for HIV/syphilis and the presence of genital human papillomavirus (HPV) for model 2. The results were adjusted for socioeconomic variables.

**Results:**

The prevalence of PSOLs was 76% higher among individuals who had two or more sexual partners in the past year (*p* = 0.046) and 68% higher in those who reported not using condoms for contraception (*p* = 0.032). The group with HIV/syphilis or self-reported STI had a 140% higher PSOL prevalence (*p* = 0.003).

**Conclusions:**

The self-report of oral lesions in adolescents and young adults may suggest risky sexual behavior and the presence of STI.

**Clinical relevance:**

It is necessary to contextualize the reality of the young person to optimize oral health care.

## Introduction

Persistent oral lesions lasting more than 15 days may have various etiologies, including trauma-related, autoimmune, and infectious etiologies. Infectious lesions have a broad and diverse etiological spectrum and include endodontic and periodontal lesions, such as abscesses and fistulas of a fungal origin, including candidiasis, and oral manifestations of sexually transmitted diseases, such as oral lesions caused by syphilis [1] and those caused by the human papillomavirus (HPV), including condyloma acuminatum, which most commonly affects adolescents and young adults [2].

Specifically, the role of HPV in the etiology of oral lesions began to gain prominence based on evidence associating HPV with the development of oral and oropharynx cancer. HPV is currently considered the most important risk factor for oropharyngeal cancer [3, 4]. HPV-associated neoplasms exhibit clinical and epidemiological characteristics different from those of neoplasms that are not associated with HPV, including the profile of individuals affected: These patients are predominantly younger white men and often do not have a history of smoking or alcohol consumption [5].

Given these and other discrepancies, HPV-related oropharyngeal cancer is considered a clinical and epidemiological entity distinct from oropharyngeal cancer unrelated to HPV [3], thus requiring different prevention strategies. Accordingly, oral HPV infection is notably associated with aspects of sexual behavior: It is less prevalent in individuals with no history of sexual contact, and its occurrence increases proportionally with the number of sexual partners [6]. Moreover, the practice of oral sex seems to be associated with an increased risk of infection in young individuals [7, 8].

The increasing participation of HPV in the etiology of oropharyngeal cancer and the pattern identified among younger individuals suggests an association of the disease with sexual behavior [8]. Testing this hypothesis, Farsi et al. (2015) analyzed the relationship between sexual behavior aspects and the risk of developing head and neck cancer in a systematic literature review with a meta-analysis and identified a statistically significant association between the number of sexual partners and an increase in this risk (odds ratio (OR) = 1.29, 95% confidence interval (CI_95_) = 1.02 to 1.63 for the comparison between the most extreme categories of this variable) [9]. Furthermore, the association of HPV with cancerous lesions of the oral mucosa, such as leukoplakias and erythroplasias, has also been demonstrated [10–12].

These findings reinforce the understanding that oral health and sexual and reproductive health are often linked, and dental teams can therefore participate more actively in this interface, reflecting a very valuable opportunity especially for the young population, which has a high prevalence of sexually transmitted infections (STI). In Brazil, approximately 15% of reported cases of AIDS between 2000 and 2018 were concentrated in the age group of 25 to 29 years [13].

With the widespread availability of the rapid test for HIV and other STI, the role of dentists in the stages of preventive counseling and in the diagnosis of these diseases has become more prominent [14, 15]; however, the dental environment remains underutilized for this purpose [14, 16, 17]. Many aspects of the oral health/sexual and reproductive health relationship can be better understood to enhance professionals’ performance and oral health in this context, especially with regard to prevention.

The importance of identifying individuals at risk for STI through signs, symptoms, or reports addressed during dental visits has been recognized in the literature, but research has primarily focused on the diagnosis of already established conditions rather than disease prevention [18–20]. Therefore, the present study aims to investigate whether the presence of persistent self-reported oral lesions (PSOLs) is associated with aspects of sexual behavior in a sample of individuals aged 16 to 25 years who use primary health-care services in the state capitals of Brazil; this sample includes the same population that participated in the POP-Brazil study. The hypothesis of the present study, which has not been previously explored in the literature, is that the presence of PSOLs is associated with less safe sexual behaviors.

## Materials and methods

This study analyzed the data collected in the POP-Brazil study, which included a sample of individuals aged 16 to 25 years who use primary health-care services in the State capitals and the Federal District of Brazil. The study sample was calculated to detect a difference of 5% in the HPV prevalence among the macroregions of the country, with a power of 80% and an alpha of 5%, considering an HPV prevalence of 30% in Brazil—estimated by a systematic review that analyzed infection in the cervix [21]. The sample size was 1587 individuals in each of the 5 macroregions (a total of 7935). The exclusion criteria were pregnancy, having given birth within 3 months, having already undergone hysterectomy or trachelectomy or having already presented with grade 2 or more severe cervical intraepithelial neoplasia. Individuals were recruited through their reference health units, which were selected using criteria related to the representativeness of the health districts of each capital and the ability to collect and store oral samples. The POP-Brazil study team conducted an interview—based on a structured questionnaire—with each participant, collected samples from the oral cavity and cervical or penile/scrotal sites, and applied rapid tests for the diagnosis of HIV and syphilis. The methodology of the POP-Brazil study is described in detail by Wendland et al. [22]. Participants who did not complete the questionnaire, participants who answered the questionnaire but did not provide genital samples, and participants who had already received the HPV vaccine were excluded from the analysis of the present study—we excluded vaccinated individuals because it was a very small group; it was not possible to use them as a control group.

For a descriptive analysis of the sample, percentages were used for categorical variables, and the means were used for continuous variables, which were both followed by corresponding 95% confidence intervals [95%CI]. The descriptive variables included sociodemographic data (sex, race/color, family income, education, and social class), sexual behavior data (the number of partners in the past year, the type of intercourse in the last 5 years, the practice of oral sex, condom use to avoid pregnancy, condom use during the last intercourse, age at first sexual intercourse, partner’s age at first sexual intercourse, and age at first pregnancy), the self-reported history of STI (the presence or a history of syphilis, gonorrhea, herpes, condyloma, HPV, and/or some STI), tests performed by the POP-Brazil project (the rapid HIV test, the rapid test for syphilis, the presence of genital HPV, and the presence of high-risk HPV, which is related to the development of cancer), and the history of PSOLs (the presence or a history of oral lesions for more than 15 days, which the participant indicated in the questionnaire; in cases of a positive response, the anatomical location was indicated: the external part of the lips, inner part of the lips, tongue, mouth floor, inner cheek, palate, and gingiva). Box 1 shows the categorization of exposure and outcome variables and the collection format.

The percentages of all variables were calculated after applying a weighting variable to the sample, which was constructed according to the distributions of the populations of Brazilian capitals by sex in the age group of interest in 2010 according to the Demographic Census of the Brazilian Institute of Geography and Statistics (IBGE).

To analyze the associations between the presence of PSOLs (outcome variable, “yes” or “no” categories) and the other categorical variables, the chi-square test was used with a significance level of 5%. For the quantitative variables, which included age at first sexual intercourse, partner’s age at first sexual intercourse and age at first pregnancy, Student’s *t* test was used to compare the means between the 2 outcome categories, and *p* < 0.05 indicated a statistically significant result.

In the next step, 2 multivariate models were constructed to analyze the associations with the outcome variable, which provided a prevalence ratio (PR) as a measure of association. The PR was estimated by Poisson regression with robust variance. Both models included the presence of PSOLs (“yes” or “no” categories) as their outcome. In the first model, the exposure variables were age at first sexual intercourse (1-year increments), the number of partners in the past year [reference category (RC): 0 or 1 partner in the past year], oral sex practice (RC: not practicing), condom use to avoid pregnancy (RC: use), and condom use during the last intercourse (RC: used). This analysis was also stratified by sex. In the second model, the exposure variables were the presence of a self-reported STI, a positive rapid HIV test or a positive rapid test for syphilis (RC: no), and the presence of HPV in a genital sample (RC: no). This analysis was also stratified by the practice of oral sex. Both models were adjusted for sex, education, and social class.

Notably, despite having a single outcome, the 2 models were implemented because they analyzed intercourse with exposure variables of different phases: The first phase consisted of exposure variables related to the risk of developing STI, and the second phase investigated relationships with already established infections, i.e., an STI as a predictor of the outcome. All analyses were performed using SAS software version 9.4 (Statistical Analysis System, SAS Institute Inc., Cary, NC).

## Results

Data from 7694 patients aged 16 to 25 years who used primary health-care services in the state capitals of Brazil were analyzed, 5569 of whom were female, while 2125 were male. The distribution of individuals according to sociodemographic and socioeconomic characteristics and characteristics related to sexual behavior is shown in Tables 1 and 2, respectively. In these tables, the distribution is also presented considering the presence or absence of PSOLs.

**Table 1:** Sample distribution by sociodemographic and socioeconomic characteristics—the total frequency and considering the presence of persistent self-reported oral lesions (PSOLs) (Brazil, 2017)

Variable	% [95%CI]	Without PSOLs (%)	With PSOLs (%)	*p* value
Sex				
Female	47.85 [45.83, 49.86]	97.61	2.39	0.498
Male	52.15 [50.14, 54.17]	97.21	2.79	
Race/color				
White	23.96 [22.16, 25.76]	97.67	2.33	0.464
Black	16.76 [15.19, 18.33]	96.49	3.51	
Brown	56.78 [54.72, 58.85]	97.49	2.51	
Other	2.49 [1.77, 3.22]	98.47	1.53	
Family income^b^				
Less than BRL 830	22.42 [20.49, 24.35]	96.27	3.73	0.047
Between BRL 830 and BRL 1659	19.05 [17.39, 20.71]	96.82	3.18	
Between BRL 1660 and BRL 2489	39.93 [37.85, 42.01]	98.18	1.82	
Above BRL 2490	18.60 [16.90, 20.30]	98.26	1.74	
Education^c^				
Illiterate/elementary	23.52 [21.72, 25.32]	97.08	2.92	0.443
Secondary school	55.09 [53.03, 57.14]	97.75	2.25	
Higher education	21.39 [19.74, 23.04]	96.85	3.15	
Social class				
A–B	17.92 [16.32, 19.51]	96.81	3.19	0.309
C	56.08 [54.05, 58.12]	97.84	2.16	
D–E	26.00 [24.27, 27.73]	96.86	3.14	
Mean [95%CI]				
Age	21.57 [21.46, 21.68]	21.56	21.94	0.049

^b^Value in Brazilian Reais (BRL); ^c^levels of education consider complete or incomplete elementary education, complete or incomplete secondary education, and complete or incomplete higher education

**Table 2:** Sample distribution by sexual behavioral characteristics—the total frequency and considering the presence of persistent self-reported oral lesions (PSOLs) (Brazil, 2017)

Variable	% [95%CI]	Without PSOLs (%)	With PSOLs (%)	*p* value
No. of partners—past year				
Fewer than two	67.01 [64.93, 69.08]	97.69	2.31	0.245
Two or more	32.99 [30.92, 35.07]	96.93	3.07	
Type of intercourse—last 5 years				
Opposite sex	92.38 [91.19, 93.56]	97.81	2.19	0.028
Both or same sex	7.62 [6.44, 8.81]	94.45	5.55	
Oral sex practice				
No	28.15 [26.31, 29.99]	97.04	2.96	0.308
Yes	71.85 [70.01, 73.69]	97.72	2.28	
Condom use to avoid pregnancy				
No	49.29 [47.21, 51.37]	97.18	2.82	0.503
Yes	50.71 [48.64, 52.79]	97.61	2.39	
Condom use during the last intercourse				
No	59.84 [57.82, 61.87]	97.51	2.49	0.644
Yes	40.16 [38.13, 42.18]	97.20	2.80	
Mean [95%CI]				
Age at first intercourse	15.21 [15.11, 15.30]	15.20	15.46	0.113
Partner’s age at first intercourse	18.47 [18.27, 18.67]	18.44	19.58	0.005
Age at 1st gestation	17.06 [16.82, 17.30]	17.07	16.57	0.138

Of the individuals interviewed who answered the question about PSOLs (7693), 2.60% [1.98, 3.22] reported having or previously having some lesion that remained in the mouth for 15 or more days. The most frequently reported lesion site was “the outside of the lips” (43.34% [31.11, 55.57]), followed by “the inner part of the lips” (24.72% [13.89, 35.54]); these results are shown in Table 3.

**Table 3:** Sample distribution by the presence and location of persistent self-reported oral lesions (PSOLs) (Brazil, 2017)

	No (%[95%CI])	Yes (%[95%CI])
Persistent Oral Lesion	97.40 [96.78, 98.02]	2.60 [1.98, 3.22]
Location		
External part of the lips	–	43.34 [31.11, 55.57]
Internal part of the lips	–	24.72 [13.89, 35.54]
Internal part of the cheeks	–	19.47 [10.88, 28.06]
Tongue	–	19.27 [10.93, 27.61]
Mouth floor	–	9.45 [2.38, 16.53]
Gingiva	–	9.42 [3.50, 15.34]
Palate	–	5.23 [0.49, 9.98]

A statistically significant difference was observed in the PSOL frequency between the family income categories (*p* = 0.047) and the mean age of the individuals (*p* = 0.049). The relative frequency of PSOLs was higher in the lower-income categories, and the mean age was higher among individuals who reported PSOLs (Table 1). Regarding the variables of sexual behavior, a statistically significant difference was observed in the frequency of PSOLs between the intercourse types in the last 5 years (*p* = 0.028) and the mean ages of the first sexual intercourse partner (*p* = 0.005). The relative frequency of PSOLs was higher among individuals who reported having sexual intercourse with individuals of the same sex or both sexes in the last 5 years than that among the participants who reported having intercourse with people of the opposite sex in the last 5 years. The mean age of the first sexual intercourse partner was higher among the individuals who reported PSOLs (Table 2).

Approximately 12.74% [11.38, 14.10] of the sample reported having or previously having some type of STI. These individuals had a higher frequency of PSOLs, as shown in Table 4, which also shows the distribution in relation to the type of self-reported STI, the results of the rapid tests for HIV and syphilis, and the results of sample collection for genital HPV detection. The frequency of PSOLs was not statistically different between individuals with and without genital HPV (*p* = 0.594).

**Table 4:** Distribution of the sample by the presence of sexually transmitted infections (STI)—the total frequency and considering the presence of persistent self-reported oral lesions (PSOLs) (Brazil, 2017)

STI	% [95%CI]	Without PSOLs (%)	With PSOLs (%)	*p* value
Syphilis^a^				
No	97.06 [96.44, 97.67]	97.51	2.49	0.085
Yes	2.94 [2.33, 3.56]	95.05	4.95	
Gonorrhea ^a^				
No	96.03 [95.05, 97.02]	97.53	2.47	0.141
Yes	3.97 [2.98, 4.95]	95.01	4.99	
Herpes^a^				
No	97.96 [97.41, 98.51]	97.40	2.60	0.049
Yes	2.04 [1.49, 2.59]	98.98	1.02	
Condyloma^a^				
No	96.75 [96.17, 97.33]	97.60	2.40	0.003
Yes	3.25 [2.67, 3.83]	92.52	7.48	
HPV^a^				
No	96.88 [96.26, 97.51]	97.54	2.46	0.025
Yes	3.12 [2.49, 3.74]	93.78	6.22	
Some STI^b^				
No	87.26 [85.90, 88.62]	97.78	2.22	0.004
Yes	12.74 [11.38, 14.10]	95.13	4.87	
Rapid HIV test				
Negative	98.02 [97.07, 98.98]	96.78	3.22	–
Positive	1.14 [0.25, 2.03]	100.00	0.00	
Fast test for syphilis				
Negative	94.27 [92.66, 95.88]	96.93	3.07	0.664
Positive	5.36 [3.76, 6.96]	95.52	4.48	
Genital HPV				
Absent	46.40 [44.21, 48.59]	97.38	2.62	0.594
Present	53.60 [51.41, 55.79]	96.98	3.02	
High-risk HPV				
Absent	64.81 [62.76, 66.86]	97.25	2.75	0.752
Present	35.19 [33.14, 37.24]	97.00	3.00	

^a^The self-reported situation referring to a patient’s past and present condition. ^b^The self-reported situation referring to a patient’s past and present condition, including the STI “syphilis,” “gonorrhea,” “herpes,” “condyloma,” “HPV,” and “other”

Regarding the analysis of associations between PSOLs and sexual behavioral variables, including age at first sexual intercourse, the number of partners in the past year, the practice of oral sex, condom use to avoid pregnancy, and condom use during the last intercourse, the prevalence of these lesions was found to be 76% higher among individuals who had 2 or more sexual partners in the past year than that among individuals who had fewer than 2 partners (*p* = 0.046). In addition, individuals who reported not using condoms as a contraceptive method had a 68% higher prevalence of self-reported oral lesions than that among those who reported using condoms (*p* = 0.032). These results are independent of sex, education level, social class, and other behavioral variables that constituted the model. The other variables analyzed did not show statistically significant associations with the outcome, and these results are detailed in Table 5.

**Table 5:** The adjusted prevalence ratio (PR) of persistent self-reported oral lesions (PSOLs) and its relationships with the listed variables considering all individuals, only men and only women (Brazil, 2017)

Variables	Total	Men	Women
	PR [95%CI]	PR [95%CI]	PR [95%CI]
Age at first sexual intercourse	1.01 [0.92, 1.12]^a^	1.05 [0.91, 1.22]^b^	0.97 [0.86, 1.09]^b^
Two or more partners in the past year	1.76* [1.01, 3.05]^a^	1.30 [0.56, 2.98]^b^	2.44** [1.37, 4.32]^b^
Oral sex practice (yes)	0.69 [0.42, 1.16]^a^	0.89 [0.35, 2.28]^b^	0.61 [0.32, 1.13]^b^
Condoms as a contraceptive (no)	1.68* [1.05, 2.69]^a^	1.86 [0.76, 4.55]^b^	1.55* [1.00, 2.39]^b^
Condom use during the last intercourse (no)	1.06 [0.66, 1.70]^a^	1.03 [0.45, 2.35]^b^	1.07 [0.65, 1.77]^b^

^a^Result adjusted for sex, social class, and education level and by the other variables shown in the table

^b^Result adjusted by social class and education level and by the other variables shown in the table; *p < 0.05; **p < 0.01

When the analysis was stratified by sex, among men, no variable was statistically associated with the presence of PSOLs. Among women, both the number of partners in the past year and the use of condoms as a contraceptive method maintained statistically significant associations with the outcome: Women who had 2 or more partners in the past year had a 2.44-times greater prevalence of PSOLs than women who had fewer than 2 partners (*p* = 0.002), and women who reported not using condoms as a contraceptive method had a 55% higher prevalence of PSOLs than those who reported using condoms (*p* = 0.049). These results are unrelated to the education level or social class of the individuals and are detailed in Table 5.

**Fig. 1:**
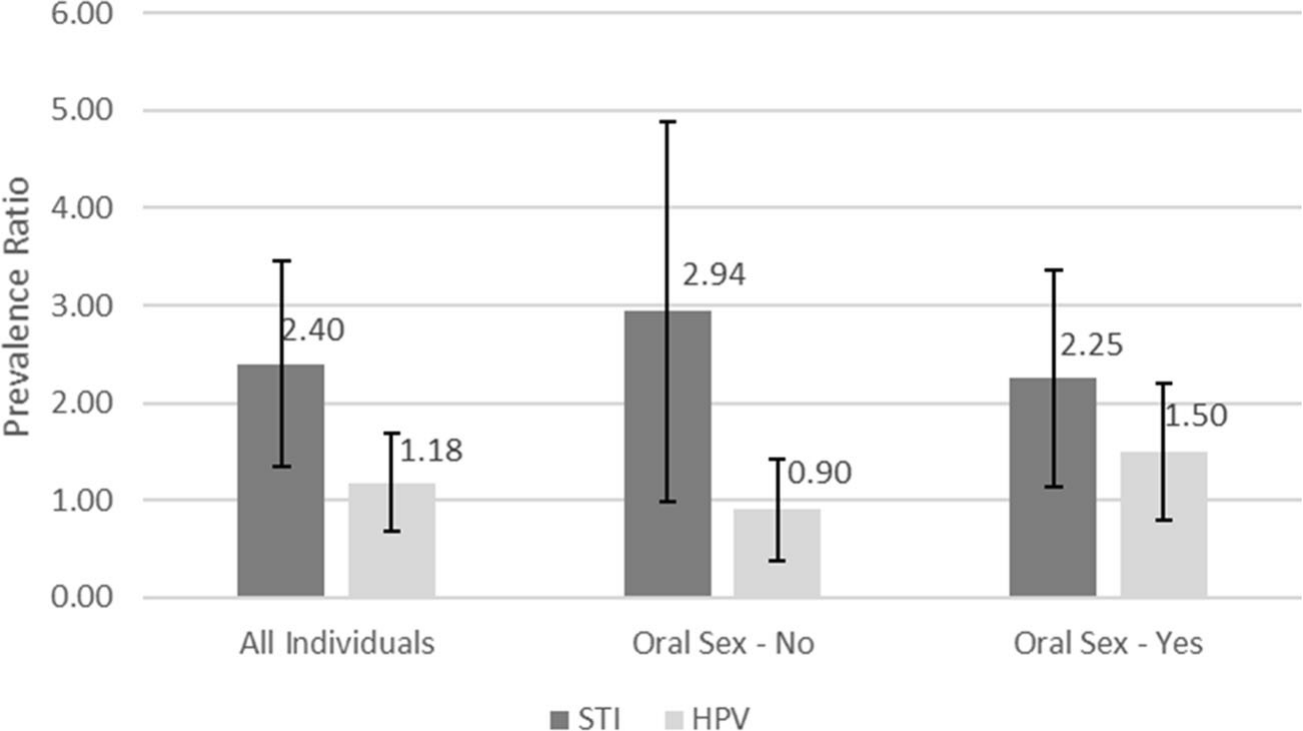
The adjusted prevalence ratio and its 95% confidence interval for persistent self-reported oral lesions (PSOLs) and its relationships with the presence of sexually transmitted infections (STI) and the presence of HPV in a genital sample (HPV) considering all individuals, only individuals not practicing oral sex, and only individuals practicing oral sex (Brazil, 2017). STI: the presence of a self-reported STI, a positive rapid HIV test or a positive rapid test for syphilis. The results have been adjusted by sex, social class, and education level

Finally, the associations between PSOLs and the presence of STI (self-reported and/or identified by rapid tests) and genital HPV were analyzed. The group of individuals diagnosed with HIV or syphilis and/or had self-reported STI had a 2.4-times [1.35, 4.27] higher prevalence of PSOLs than the group that did not have STI (*p* = 0.003). However, when the analysis was stratified by oral sex practice, the results were different. The association lost statistical significance among individuals who did not practice oral sex [prevalence ratio (PR) = 2.94 [0.99, 8.71]. Among the individuals who practiced oral sex, the statistical significance of the association persisted: The group of individuals diagnosed with HIV or syphilis and/or had self-reported STI had an approximately 125% higher prevalence of PSOLs than the group that did not present STI (*p* = 0.021). These results are independent of the education level and social class of the individuals and the presence of genital HPV and are shown in Fig. 1. No statistically significant association was identified between PSOLs and genital HPV (no stratification, PR = 1.18 [0.68, 2.04]; no oral sex practice, PR = 0.90 [0.37, 2.23]; oral sex practice, PR = 1.50 [0.81, 2.81]).

## Discussion

The main results of this study indicate associations between PSOLs and aspects related to the sexual behavior of young people, suggesting that some behavioral patterns with a higher risk for the development of STI are also related to the presence of PSOLs. The identification of this relationship, which has not yet been addressed in the literature, is a helpful alert to health professionals involved in the care of young people for the prevention and early diagnosis of STI. Besides, this can be an indication that dental consultations represent a valuable opportunity for a comprehensive approach to these individuals with an emphasis on aspects related to their sexual and reproductive health.

The relationship between oral health and sexual and reproductive health is recognized in the literature, which focuses on the characterization of oral lesions resulting from already established STI and their role as markers for the diagnosis or progression of these infections. The most illustrative examples of this relationship are the oral manifestations of AIDS, such as oral candidiasis, oral hairy leukoplakia and periodontal disease [19, 23, 24], and oral lesions caused by acquired syphilis [1, 20, 25]. The oral manifestations related to these disorders may even be the first symptoms of infection or disease progression, which reinforces the importance of their identification during dental consultations [18].

In the present study, patients with self-reported or diagnosed STI significantly more frequently reported having PSOLs according to both the results of the simple analysis (Table 4) and the results of the model adjusted for sex, social class, and education level (Fig. 1). Given that the etiologies of the self-reported lesions were not known, the association found is susceptible to different interpretations. This relationship may reflect a pattern of neglect towards health-related self-care (or lack of access to such care) as oral lesions may be due to, for example, dental infections. A second interpretation is the existence of a clinical relationship between the presence of STI and oral lesions, which would be compatible with the stratification results in the adjusted model (Fig. 1): The association between an HIV or syphilis diagnosis or a self-reported STI and PSOLs lost significance among individuals who did not practice oral sex but persisted among those who practiced oral sex, which increases the oral inoculation of sexually transmitted pathogens [26]. Regardless of the interpretation, self-reports of PSOLs may serve as strategic information to assist in the early diagnosis of STI.

With the identification of the growing role of HPV in the etiology of head and neck cancer, aspects of the sexual behavior of patients began to be investigated [27]. Greater numbers of sexual partners and oral sex partners seem to be associated with an increased risk of developing head and neck cancer (OR = 1.29, 95% CI = 1.02 to 1.63 and OR = 1.69, 95% CI = 1.00 to 2.84, respectively) [9]. Notably, specifically in oral cancer, the association with exposure variables of this nature has not been demonstrated [28]. The establishment of this relationship introduced a new responsibility to professionals in the field of head and neck cancer involving counseling regarding sexual behavior rather than only traditional risk factors such as smoking and alcohol consumption [27]. However, the approach of the oral health and sexual behavior interface towards prevention remains minimally explored.

In the analysis adjusted for sex, social class, and education level in this study (Table 5), not using condoms as a contraceptive method and having intercourse with 2 or more partners in the past year were identified as behaviors significantly associated with a higher prevalence of PSOLs. Both behaviors are considered risky practices for the development of STI [29] and had high sample frequencies (49.29% and 32.99%, respectively). Given these results, information on the presence of PSOLs obtained during dental consultations with adolescent and young adult patients or from collective approaches in the general population can be powerful to facilitate a dialog regarding safe sex practices and STI prevention, early diagnosis of related conditions, and the provision of comprehensive and multidisciplinary care to these individuals.

The adolescent patient health approach should consider the peculiarities of this life stage. At this stage, neurophysiological maturation is still incomplete, and the ability to understand the relationship between behavior and consequences is not fully developed. These individuals are often exposed to some pressure towards experimentation, which includes drug use and early sexual contact [30]. In addition, adolescents and young adults usually seek health services for acute and specific complaints and not for health counseling and/or reviews [31, 32] and are concerned with confidentiality regarding their relationship with the health service [33]. This population (15 to 24 years old) bears the greatest STI burden among all age groups, accounting for half of the 20 million new cases diagnosed annually in the USA [34]. Thus, general dentists and dental hygienists are often the only health professionals involved in this scenario. A study of 743 adolescents in Southern Brazil indicated that “health problems” were the main reason (69.0%) for them to seek health care in the last month. When researchers detailed these problems, they identified oral health as the second most frequent complaint, second only to respiratory issues [31]. The latest National Survey of Oral Health in Brazil indicated that 85.8% of adolescents aged 15 to 19 consulted a dentist at some time in their lives, and 53.9% of these consulted less than 1 year ago—despite regional discrepancies, these totals were never less than 79.8% and 50%, respectively [35]. The results in the 2013 National Health Survey of Brazil (PNS) indicated similar percentages as follows: 51% of young people aged 18 to 29 years visited a dentist in the last 12 months [36].

The willingness of oral health teams to address sexual health issues is a topic that requires attention. The technical-centered profile of dentistry, aggravated by the fact that sexual health is a sensitive question, can result in the underutilization of dental consultation, which could be an opportunity for comprehensive care for youths. A study carried out with 929 dentists from Florida/USA indicated that these professionals express less intention to advise adolescents about HPV—compared with counseling on sugar consumption—mainly due to the perception that this is less socially acceptable conduct [37]. In another study, dental providers—dentists and dental hygienists—reported the lack of ability to conduct this type of conversation as a barrier to acting in the education for HPV prevention; they also related concerns about the reaction of patients (or their parents) and the scarcity of time and privacy in the care spaces [38]. However, dentists are the professionals who most know about changes in the oral mucosa and the dental hygienists can be considered as “prevention experts.” Due to these privileged and opportune positions for counseling, further training on the topic and development of health literacy skills can be alternatives for the engagement of dental providers in prevention related to STI [39].

Moreover, coping with STI is a relevant issue in Brazil, and statistics reflecting, for example, the increased number of cases of acquired syphilis, syphilis in pregnant women, and congenital syphilis have been highlighted in the last decade. From 2016 to 2017, increases of 31.8% in the incidence of acquired syphilis, 28.5% in the syphilis detection rate among pregnant women, and 16.4% in the incidence rate of acquired syphilis (rates of 58.1, 17.2, and 8.8 cases per 100,000 inhabitants in 2017, respectively) were documented [40]. In addition, from 2014 to 2018, only 20.1% of boys aged 11 to 14 years were vaccinated against the HPV virus, although the target for the country was 80% [41]. The subtypes of this virus classified as high risk due to their roles as etiological factors of cervical, oropharyngeal, and penile cancer [4] were present in 35.19% of the sample.

The main limitation of the present study was the self-reported format of the outcome variable and some exposure variables, which did not promote the reliability of the information or clarification of the etiologies of the PSOLs. A study carried out in Southern Brazil, in which 720 young adults from a birth cohort were examined, identified papules/nodules as the most frequent alteration of the oral mucosa (32% of individuals with lesion), followed by ulcers (26.5%); the most affected location was gingiva (35%), followed by lips (21%) [42]. A 10-year review of biopsy results in oral lesions of children and adolescents found mucocele as the most common diagnosis (33.3%) [43]. As we have not found studies that validate the self-report of oral lesions, discussing the etiology of the PSOLs in this study is not very conceptual. However, we understand that there may be a consistent presence of lesions due to recurrent aphthous ulcerations, mucocele, and recurrent herpes labialis, based on the age of individuals and most common anatomical locations (Table 4) [42–44].

We emphasize that, despite being a significant limitation, self-reported oral health status is an outcome often used in population-based surveys and in studies with large samples [45], the validity of which has been demonstrated mainly in studies analyzing the periodontal conditions of individuals [46]. This strategy enables the collection of health information in unfavorable contexts for individual tests and determination of a patient’s history and not only his/her condition at a given time [44].

We found associations between PSOLs and aspects related to the sexual behavior of adolescents and young adults; some behavioral patterns—with a higher risk for the development of STI—and history or diagnosis of STI are related to the presence of PSOLs. Our results instigate some final reflections: Despite seeking to raise awareness for objectives different from those presented in the literature, such as screening for oral cancer, the results suggest the prior knowledge that mucosal examinations should be a routine practice in dental consultations. Notably, a dental anamnesis addressing oral lesion history can also be a powerful tool for the care of adolescents and young adults, allowing extension beyond the dental approach. In addition to oral diagnoses, oral health teams should be able to address issues related to the diagnosis of STI and safe sex practices, which is a critical issue mainly for young individuals who rarely attend health services and whose only link with health services is the oral health team. The reverse perspective must also be considered: All health professionals involved in the care of these patients should be aware of opportunities for oral health care, especially symptoms and signs of oral mucosal changes. The results found in the present study are powerful for promoting a more integrated, vigilant, and diligent approach to sexual and reproductive health and its interface with oral health.
